# Role of Dual-Acquisition Noninvasive Cardiac CT Imaging for the Detection of Vasospastic Angina

**DOI:** 10.3390/jcm12113753

**Published:** 2023-05-29

**Authors:** Xuan Jin, Eun-Ju Kang, Cai-De Jin, Kwang-Min Lee, Kyung-Hee Lim, Seung-Woon Rha, Cheol-Ung Choi, Hwan-Seok Yong, Sung-Cheol Yun, Matthew J. Budoff, Long-Hao Yu, Moo-Hyun Kim

**Affiliations:** 1 Department of Cardiology, Dong-A University Hospital, Busan 49201, Republic of Korea; xuan880819@126.com (X.J.); jincaide1118@163.com (C.-D.J.); tnt849@hanmail.net (K.-M.L.);; 2 Department of Cardiology, Affiliated Hospital of Yanbian University, Yanji 133099, China; 3 Department of Radiology, Dong-A University Hospital, Busan 49201, Republic of Korea; medcarrot@dau.ac.kr; 4 Department of Cardiology, Affiliated Hospital of Zunyi Medical University, Zunyi 563003, China; 5 Department of Cardiology, Korea University Guro Hospital, Seoul 08308, Republic of Korea; swrha617@yahoo.co.kr (S.-W.R.);; 6 Department of Radiology, Korea University Guro Hospital, Seoul 08308, Republic of Korea; 7 Department of Clinical Epidemiology and Biostatistics, University of Ulsan College of Medicine, Asan Medical Center, Seoul 05505, Republic of Korea; 8 Department of Medicine, Lundquist Institute, Harbor-UCLA Medical Center, 1124 W Carson Street, Torrance, CA 90502, USA; 9 Department of Cardiology, Affiliated Hospital of Guilin University, Guilin 541001, China

**Keywords:** coronary vasospasm, computed tomography angiography, specificity, positive predictive value

## Abstract

Background: Vasospastic angina (VSA) is characterized by chest pain at rest with transient ischemic electrocardiographic changes in the ST segment, and a prompt response to nitrates. Vasospastic angina is among the most frequent of the coronary artery diseases in Asia, and coronary computed tomography angiography (CCTA) may become available as a non-invasive diagnosis method. Methods: We prospectively enrolled 100 patients with suspected vasospastic angina at two centers from 2018 to 2020. All patients underwent baseline CCTA without a vasodilator in the early morning followed by catheterized coronary angiography and spasm testing. CCTA with intravenous infusion of nitrate (IV) was repeated within 2 weeks of baseline CCTA. Vasospastic angina as detected by CCTA was defined as significant stenosis (≥50%) with negative remodeling without definite plaques or diffuse small diameter (<2 mm) of a major coronary artery with a beaded appearance on baseline CT that completely dilated on IV nitrate CT. We analyzed diagnostic performance of dual-acquisition CCTA for the detection of vasospastic angina. Results: The patients were categorized into three groups according to their provocation test result (negative, *n* = 36; probable positive, *n* = 18; positive, *n* = 31). The diagnostic accuracy in terms of CCTA per patient had a sensitivity of 55% (95% CI, 40–69), specificity of 89% (95% CI, 74–97), positive predictive value (PPV) of 87% (95% CI, 72–95), and negative predictive value (NPV) of 59% (95% CI, 51–67). Conclusions: Dual-acquisition CCTA can support the non-invasive detection of vasospastic angina with relatively good specificity and PPV. CCTA was helpful for non-invasive screening of variant angina.

## 1. Introduction

Vasospastic angina is among the most frequent of the coronary artery diseases in Asia [1], and is characterized by ST elevation during angina attacks. According to the guidelines of the Japanese Society of Cardiology (JCS) published in 2013, the coronary spasm provocation test is a standard diagnostic method [2]. In addition to coronary angiography with provocation testing, noninvasive ergonovine stress echocardiography is used to discharge patients. However, these imaging tests can lead to severe myocardial ischemia, fatal arrhythmia, or even cardiac death [3,4]. Recently, coronary computed tomography angiography (CCTA), which can accurately evaluate and diagnose coronary artery stenosis, has become available as a non-invasive imaging method for coronary artery disease [5]. However, very few studies on the diagnostic value and imaging data for CCTA diagnosis of vasospastic angina have been conducted.

The basal coronary tone is augmented during periods of active vasospasm on both spastic and adjacent segments and was restored to normal during disease inactivity [6]. Previously, our study group reported a pilot study conducted in 21 patients with highly suspected variant angina, and the results supported the possibility for diagnostic usefulness with dual-acquisition of CCTA (baseline CT without vasodilator and intravenous nitrate injected CT) with a sensitivity of 73% and specificity of 100% [7]. Positive criteria for VSA on CCTA in the above study were defined as significant stenosis with negative remodeling but no definite evidence of plaques on baseline CT in an artery that completely dilated on IV nitrate CT, or diffuse small diameter (<2 mm) of a major coronary artery with a beaded appearance on baseline CT that completely dilated on IV nitrate CT. Previous pilot studies have been limited by small sample sizes.

The present study sought to investigate the feasibility of a dual-acquisition CCTA protocol for the noninvasive detection of coronary spasm in patients with suspected vasospastic angina. We assessed the diagnostic performance in prospective multicenter subjects relative to the gold standard invasive testing.

## 2. Methods

### 2.1. Study Design and Patient Population

In this prospective study conducted at two centers, 100 patients with suspected vasospastic angina were screened from 2018 to 2020 (Dong-A University Hospital, *n* = 80; Korea University Guro Hospital; *n* = 20). Our spasm registry excluded patients with coronary stents, a history of cardiomyopathy, valvular heart disease, stroke, or renal insufficiency (serum creatinine > 2.5 mg/dL). Eligible subjects then underwent “baseline CT” (initial CT without a vasodilator) between 7 and 8 a.m., followed by “IV nitrate CT” with intravenous (IV) infusion of nitrate repeated within 2 weeks of baseline CT. Catheterized coronary angiography (CAG) and ergonovine (EG) spasm provocation testing were performed on the same day or different days of CCTA. The sequence for the CCTA and EG spasm tests was adjusted based on the circumstances. The results of dual-acquisition CCTA images were compared with the spasm provocation test by independent reviewers. The incidence of major cardiovascular incidents was assessed for 9 months after the test was analyzed. The studies involving human participants were reviewed and approved by the Institutional Ethics Committee of Dong-A University Hospital (protocol code: DAUHIRB-17-114, approval: May 2018). The NAVIGATOR study has been registered at https://www.clinicaltrials.gov (NCT03570671). The patients/participants provided their written informed consent to participate in this study.

### 2.2. Coronary Angiography and Spasm Provocation Test

All patients were free from vasodilator agents for at least 3 days before the EG provocation test. The details of the techniques have been previously reported [8]. Briefly, angiography was performed by experienced cardiologists via the transradial or transfemoral approach, applying a hydrophilic sheath without intra-arterial administration of verapamil or nitroglycerin. A single coronary diagnostic catheter (Tiger Catheter, Terumo, Tokyo, Japan) was placed into the left coronary artery (LCA) and then the right coronary artery (RCA). If insignificant stenosis (<50% diameter stenosis) was found, intracoronary provocation was performed with EG (10–20 μg) injected into each coronary artery at 1 min intervals for a total of 30–50 in the RCA and 40–60 in the LCA. Usually, the RCA test was completed first, followed by LCA. If a spasm was provoked, intra-coronary nitroglycerin was immediately injected for spasm relief. Before finishing the procedure, intracoronary nitroglycerin (100 μg) and amlodipine (5 mg) were administered to prevent delayed coronary spasms, even if a spasm had not been provoked during EG administration. A positive test result was defined as transient total or sub-total coronary artery occlusion (>90% constriction) with or without angina and ischemic ECG change [9]. The probable positives were defined as insignificant coronary artery occlusion (50–90% constriction) with or without angina. The Japanese Coronary Spasm Association (JCSA) scoring system was used for risk assessments and prognostic stratification for VSA patients [10].

### 2.3. CCTA Acquisition and Analysis

All CCTA images were carefully reviewed by two independent investigators who were blinded to the patients’ clinical information. The overall agreement per patient and per vessel were 0.781 (0.601–0.960) and 0.759 (0.618–0.901).

All CCTA images were scanned using a 320-detector row CT scanner (Aquilion ONE, Canon Medical Systems, Otawara, Japan) in both medical centers with the same imaging acquisition protocol. In brief, a bolus of 50–60 mL nonionic contrast material (Ultravist^®^; Bayer AG, Berlin, Germany) was injected intravenously at a rate of 4 mL/s followed by 30 mL of contrast-saline mixture (2:8 dilution) at the same speed. The CT scans were obtained by automatic bolus tracking in the ascending aorta with a delay of 5 s (the threshold level was 100 Hounsfield units). All coronary arteries greater than 1.5 mm in diameter were analyzed by CCTA. All images were transferred to commercial software (Aquarius iNtuition Edition v4.4.11, TeraRecon Inc., Foster City, CA, USA) and analyzed by two experienced radiologists.

**Figure 1:**
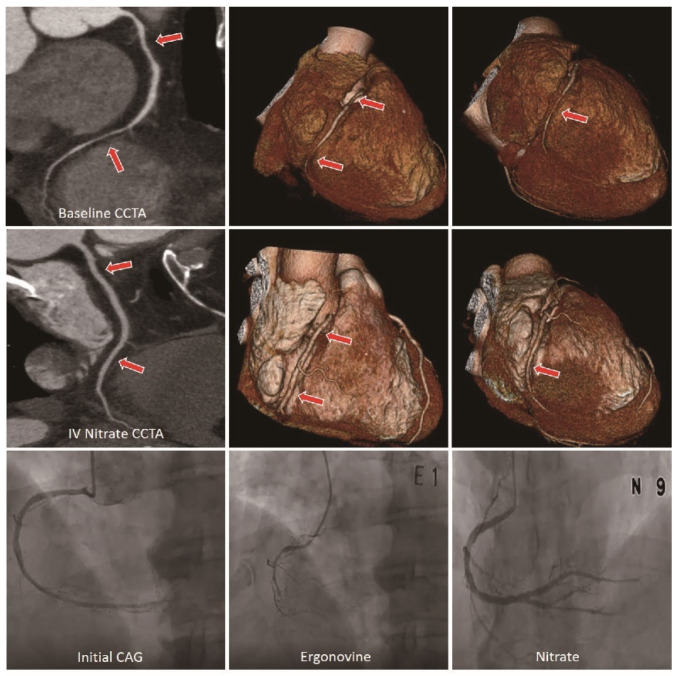
Representative case of a patient with vasospastic angina. E, ergonovine; IV, intravascular; N, nitrate.

We analyzed multiplanar reconstruction and axial thin section images of each of the coronary arteries for baseline CT and IV nitrate CT. In CCTA, the positive criteria for coronary spasms were defined as follows: (1) when the lesion was completely dilated on IV nitrate CT with more than 50% significant stenosis and negative remodeling on baseline CT, and no clear evidence of plaque, (Supplementary Figure S1) or; (2) when there was a diffuse small diameter (less than 2 mm) of the main coronary artery with a beaded appearance on baseline CT and the lesion was completely dilated on IV nitrate CT [5,7,11] (Supplementary Figure S2). Figure 1 describes the sample images of the double acquisition protocol for a representative vasospastic angina case.

We verified the correlation between basal coronary tone and spasm inducibility with the luminal data measured by CCTA, using the formula as follows: Basal coronary tone (%) = [(mean luminal diameter after nitrate -baseline mean luminal diameter)/baseline mean luminal diameter] × 100% [12]. For quantitative analysis of coronary arterial distensibility, we measured the cross-section area (CSA) and diameter of the coronary artery at the spasm site and the proximal and distal reference site. The reference sites were defined by visually normal-looking vessels within a 10 mm proximity to the spasm site. In the case of diffuse-type spasms, we measured three points on each coronary vessel—most stenotic spasm sites and within a 10 mm proximal and distal site to the spasm-related segment. We also analyzed the reference segments for normal individuals (spasm-negative group) on visually normal-looking vessels (for three major coronary arteries; LAD, LCX, and RCA). The measurement points for LAD, LCX, and RCA were defined as the diagonal branch, OM branch, and right ventricular branch, respectively.

The coronary vessel distensibility index (CDI) for CSA and diameter were calculated with the following formulas:
CDI − CSA= [(CSA IV nitrate − CSA initial)/CSA IV nitrate] × 100%or CDI − D= [(Diameter IV nitrate − Diameter initial)/Diameter IV nitrate] × 100%

### 2.4. Statistical Analysis

In a cardiac MDCTA study without intravenous NTG injection by Kang EJ et al. [7], MDCTA showed high specificity (100%) but low sensitivity (48%) in the diagnosis of variant angina. Based on this, the diagnostic sensitivity of the dual-acquisition of MDCTA in the present study was set at 75% to assess the diagnostic superiority of dual-acquisition of MDCTA, a non-invasive cardiac imaging test in variant angina. It was calculated with 80% power, and the dropout rate predicted that 15% of patients had severe stenosis due to CAG and 3% of patients’ CT images could not be read, with 5% of patients not being followed up. The total sample value was determined to be 100 people. The sample size was calculated with SPSS software (v 22.0, SPSS Inc., Chicago, IL, USA).

A descriptive analysis was performed by presenting data as mean values with standard deviation (SD) or n (%). Continuous variables were compared with the paired-sample *t*-test or the Wilcoxon rank-sum test, and categorical variables were compared with the χ^2^ test or the Fisher exact test. We analyzed the results of CCTA by using the EG provocation test as the reference standard (probable positives were considered as a positive result for the EG provocation test in the final analysis), and the sensitivity, specificity, positive predictive values (PPV), negative predictive values (NPV), positive likelihood ratio, and negative likelihood ratio were calculated on a per-patient, per-vessel basis. We also compared the distensibility index by presenting data as mean standard deviations (SD), as well as used the receiver operating characteristic (ROC) curve, which calculated the highest value (sensitivity + specificity − 1), to determine the optimal cut-off value of the CDI for predicting VSA. All analyses were performed using PASW statistics software (v 22.0, SPSS Inc., Chicago, IL, USA) and MedCalc statistics software (v14.8.1, Ostend, Belgium). A two-tailed *p* value of <0.05 was used to define statistical significance.

## 3. Results

### 3.1. Baseline Characteristics of the Study Population

**Figure 2:**
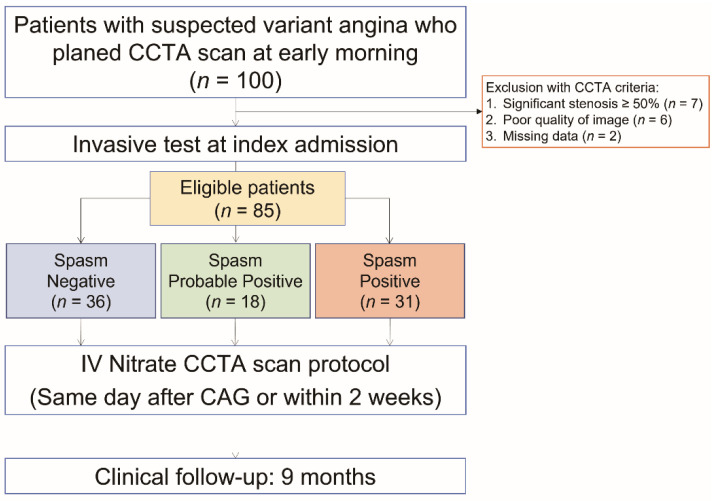
Patient selection flow diagram.

Of the 100 patients with suspected vasospastic angina, 15 patients were excluded who showed significant fixed stenosis or poor image quality, or for which there were inadequate datasets (significant stenosis ≥50%, *n* = 7; poor quality image of CCTA, *n* = 6; missing data, *n* = 2). In total, 85 patients were enrolled for the study population (Figure 2). The demographic and angiographic characteristics are summarized in Table 1. The patients were classified into three groups by EG provocation test (negative, *n* = 36; probable positive, *n* = 18; positive, *n* = 31), and all coronary provocation testing was performed without any serious complications. Among the three groups, there was no significant difference in age, with the positive group being slightly older than the other groups, as well as predominantly male. There were significantly different JCSA scores between the three groups (*p* < 0.05) with the remaining characteristics being well balanced. During 9 months of follow-up, all of the patients survived, and three patients in the positive group were re-hospitalized.

There was a higher rate of insignificant stenosis in the positive group compared to the other groups (11.1% vs. 11.1% vs. 48.4%, *p* < 0.001), as well as no significant organic stenosis observed in the three groups. CAG revealed that the spastic segments were predominantly located at the RCA (65.4%), followed by the left anterior descending (LAD) (23.1%) and left circumflex (LCX) (11.5%) arteries. Multi-vessel spasms were diagnosed in 19 patients (2 vessel, *n* = 11; 3 vessel, *n* = 8) (Table 2).

### 3.2. Reliability Prediction of CCTA Compared to Spasm Testing

All CCTAs were successfully performed without any significant complications. A total of 85 patients and 255 vessels were interpretable on CCTA, and 31 patients (36%) and 58 vessels (23%) exhibited positive results according to CCTA (Table 3). To compare the results of the CCTA and the EG spasm test, 85 patients and 197 vessels were matched. The diagnostic accuracy of CCTA per patient had a sensitivity of 55% (95% CI, 40–69), specificity of 89% (95% CI, 74–97), positive predictive value (PPV) of 87% (95% CI, 72–95), and negative predictive value (NPV) of 59% (95% CI, 51–67). In the per-vessel analysis, the sensitivity, specificity, positive predictive value, and negative predictive value of CCTA were 36% (95% CI, 25–49), 96% (95% CI, 91–99), 83% (95% CI, 66–92), and 75% (95% CI, 71–78), respectively.

In addition, JCSA scores greater than 3, syncope or CPR on admission, elevated troponin I, admission through the emergency room, and heavy drinking was associated with higher specificity and PPV (100%). Sensitivity in patients with elevated troponin (77%) or syncope or CPR (100%) was the highest (Supplementary Table S1).

### 3.3. Quantitative Analysis for Coronary Arterial Distensibility

There was a significant correlation between basal coronary tone and spasm inducibility detected by CCTA (CDI_D: R^2^ = 0.7434, *p* < 0.0001; CDI_A: R^2^ = 0.6924, *p* < 0.0001) (Supplementary Figure S3). We measured 36 patients and 36 vessels in the negative group, as well as 49 patients and 66 vessels in the positive group. The highest proportion of RCA (58.3%) was in the positive group, followed by LAD (25%) and LCX (16.7%), with the most common morphologic type of the detected spasms being the diffuse type (83.3%) (Supplementary Table S2). ROC curves were analyzed based on the results.

**Figure 3:**
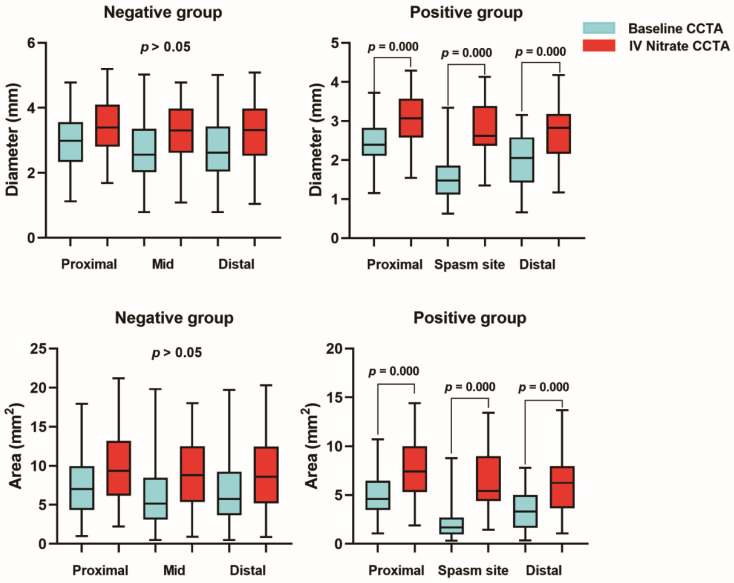
Comparison of the reference and spasm segment characteristics.

**Figure 4:**
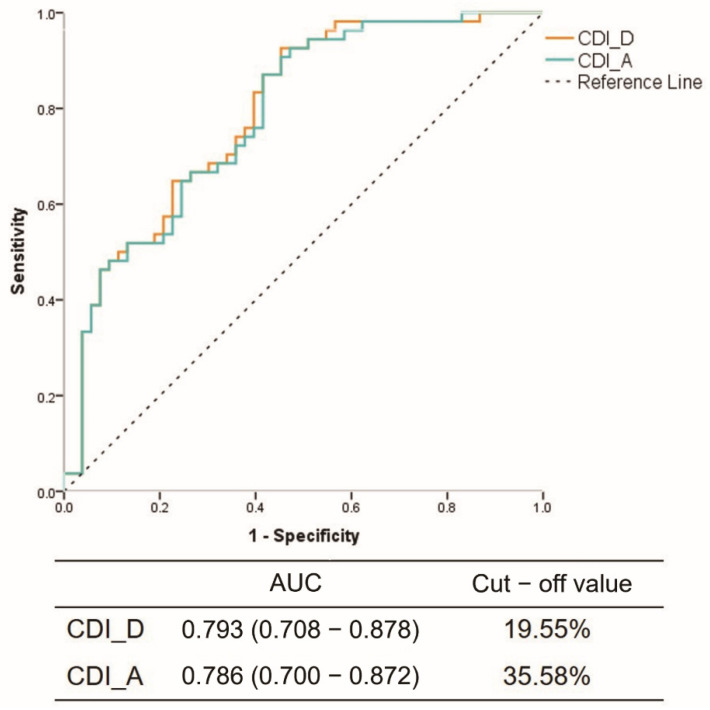
Receiver operating characteristic curves for predicting coronary vessel distensibility index by diameter (CDI-D) and coronary vessel distensibility index by area (CDI-A).

Compared with baseline CCTA, the values for diameter and area of the coronary arteries were larger in IV nitrate CT in both spasm and normal reference groups. The CSA of the reference segments were marginally increased, but there was no statistical significance. In the spasm segment, compared with baseline CCTA, the values for diameter and CSA were significantly increased at the spasm site (diameter 1.48 ± 0.56 vs. 2.60 ± 0.70; CSA 1.96 ± 1.53 vs. 5.66 ± 2.94) (*p* < 0.001) (Table 4, Figure 3). In addition, coronary distensibility in the positive group was significantly higher compared to the negative group (CDI_D: 17.7 ± 15.4 vs. 42.3 ± 17.3, *p* < 0.001; CDI_A: 31.5 ± 24.9 vs. 63.6 ± 21.0, *p* < 0.001) (Table 4). In the ROC analysis, to predict VSA, the optimal cut-off value for CDI_D was 19.55% (AUC 0.793; 95% CI 0.708–0.878) and 35.58% (AUC 0.786; 95% CI 0.700–0.872) for CDI_CSA (Figure 4).

## 4. Discussion

According to the existing JCS guidelines, the coronary provocation test has been the gold standard for the diagnosis of suspected vasospastic angina. However, the procedure is invasive and carries some risks for patients such as myocardial ischemia or infarction, serious arrhythmia, and puncture-site-related complications. Therefore, further investigation of non-invasive and safe diagnostic methods is needed.

The pathogenesis can be explained by increased myocardial oxygen demand in the presence of fixed stenosis of epicardial coronary arteries. The pathogenesis of VSA is likely multifactorial, including smooth muscle cell hyper-reactivity, endothelial cell dysfunction, and various environmental factors such as smoking, alcohol consumption and metabolic abnormalities [13]. Circadian variations in the production of various hormones including cortisol, vasopressin, melatonin, growth hormone, or inflammatory cytokines may also be associated with the circadian variation of VSA [14].

Kim et al. conducted a study using ergonovine stress echocardiography (ESE) in patients with acute coronary syndrome as angiographically normal or near-normal, with the results showing that 25 (48%) out of 52 patients developed left ventricular wall motion abnormalities after the intravenous bolus administration of ergonovine [15]. Previous studies have shown that ergonovine echocardiography can diagnose coronary vasospasm before angiography, with a sensitivity of 91% and specificity of 88%, with it being a safe and reliable diagnostic screening test for VSA [16,17]. However, ergonovine stress echocardiography requires a non-invasive treadmill test or nuclear myocardial scan to pre-exclude significant coronary stenosis [18], and there has also been a case report of cardiac arrest during ESE, which was recovered after cardiopulmonary resuscitation (CPR) [19]. Although rare, caution and close monitoring throughout the procedure is required.

CCTA has been emerging as a first-line diagnostic method for CAD with a strong basis in histopathology and strong clinical applicability driven by excellent negative predictive value [20]. In a recent retrospective study by Park et al., the results showed low sensitivity (7.5%) and moderate NPV (49%), but high specificity and PPV on a per-lesion analysis and per-patient analysis with coronary provocation testing as the reference standard [11]. Another study demonstrated that CCTA showed good diagnostic performance with high specificity and PPV, but sensitivity of 48% and NPV of 68% in the assessment of VSA [5]. In addition, the SCOT-HEART (Scottish Computed Tomography of the Heart) trial confirmed that the 2019 ESC estimates of pretest probability based on invasive coronary angiography and fractional flow reserve (FFR) were broadly similar to the prevalence observed on CCTA in the trial cohort, although it tended to underestimate the real prevalence, or alternatively, CCTA might overestimate the CAD [21]. Although these studies showed high specificity and PPV, the noted limitations included: (1) They were retrospective analyses with a small number of patients; (2) the interval between CCTA and coronary provocation testing was too long; and (3) only single CCTA results were analyzed in patients with VSA, and temporal variation of vasospasms could not be analyzed. As a prospective study, compared with previous efforts, our results showed a slight increase in sensitivity, as well as the maintenance of high PPV and NPV. Although there is some variability in the results, combined with other studies, owing to its high NPV, CCTA was suggested to be a safe gatekeeper for EG testing [18,22]. Nevertheless, a high NPV was found in most previous studies, especially in patients with a low pre-test likelihood of CAD. Other diseases can also cause chest pain, such as gastric ulcers and reflux esophagitis, so it can be intrusive to use CAG as a screening test in asymptomatic subjects due to its invasive nature. Chang et al. reported that CCTA could be an efficient initial triage tool in patients with acute chest pain with low-to-intermediate risk in emergency departments because of its high sensitivity and NPV [23]. However, cases with false positive results of CCTA in VSA have also been reported, leaving the position of CCTA in the diagnosis or exclusion of VSA in doubt [24,25]. Although false-positive cases were low, false-negative cases were relatively high in our study data.

Nonobstructive CAD included not only angina with epicardial coronary spasm but also microvascular spasm [26]. However, the coronary microcirculation was not easily visualized, while the epicardial coronary arteries were easily visualized by CAG or CCTA [27]. Due to the limitations of the evaluation of microvascular spasm in CCTA or CAG, this study only evaluated spasms in the relatively large coronary artery. Future research into non-invasive detection of microvascular spasm may be warranted.

There was a possibility that diagnoses were underestimated in the absence of provocation testing, and increasing the sensitivity would be challenging. However, the sensitivity was highest in those patients with elevated troponin (77%), syncope, or CPR (100%), and admission via the emergency room as well as heavy drinking were associated with higher specificity and PPV (100%). Therefore, the sensitivity may be increased by application in symptomatic patients with high JCSA scores, syncope or CPR on admission, and elevated troponin I [28].

Moreover, we did not routinely administer beta-blockers before CT, with the exception of beta blockers in cases where the patient had a higher heart rate (>70 beats per minute), because beta-blockers have theoretical adverse potential effects in patients with VSA [29]. Therefore, we used a short-acting vasodilatory beta-blocker (carvedilol), which has theoretical advantages over conventional beta-blockers. It might be unsafe to use beta-blockers in patients with highly suspected vasospastic angina if the use of any other vasodilating drugs, such as calcium-channel blockers, are interrupted prior to CT. However, our protocol permitted the sublingual use of nitroglycerine in cases where patients reported chest pain.

The potential indication for dual-acquisition CCTA would be patients with resting chest pain but without a history of medication or previous normal coronary angiography, especially those who visited the emergency room with or without troponin elevation. Other scenarios include resting chest pain patients in general hospitals without catheterization facilities, or with only delayed noninvasive cardiac CT imaging without IV nitrate for cases with normal or near-normal coronary arteries from catheterized coronary angiography (CAG) including false negative ergonovine (EG) spasm provocation.

In addition, CCTA may be useful to detect multi-vessel vasospasms because provocation testing does not usually include the contralateral vasospasm test after the spasm occurs on one side. In our study, the positive detection rate of coronary artery spasms detected by CCTA was slightly higher than with the EG test (25.8% vs. 22.6%), but the lack of statistical significance may be due to too few patients (Supplementary Table S3).

### Limitations

Although this was a prospective study, the number of patients involved remains small. Larger studies are needed to consolidate the current findings. There might also be a possibility of mismatch between the exact spasm sites on CCTA and those on CAG. However, we took every effort to ensure matching of the same lesions by using side branches and ostia as landmarks. In addition, since the heart activity had diastolic and systolic periods, we cannot confirm that the cardiac contractions occurred in the same period during CAG and CCTA, and a lack of bias cannot be guaranteed. Since patients were enrolled at two institutions, different systems may have resulted in two examinations not being performed on the same day. If such examinations were conducted on different days, the vasomotor tone state might be different, which may influence the diagnostic efficacy. However, the vast majority of patients received CCTA and CAG on the same day.

## 5. Conclusions

For patients with suspected variant angina, dual-acquisition CCTA can contribute to the non-invasive detection of vasospastic angina with relatively good specificity and PPV. However, due to the relatively low sensitivity, further studies are needed in larger populations or more specific patient subgroups, such as those with resting chest pain with ECG changes and/or mild enzyme elevation or high JCSA risk scores.

## Figures and Tables

**Table 1:** Baseline characteristics of the study population (categorized by EG test).

Variables	Negative (*n* = 36)	Probable Positive (*n* = 18)	Positive (*n* = 31)	*p*
Age (years)	59.6 ± 11.1	56.3 ± 9.1	60.0 ± 7.7	0.382
Sex (male), %	11 (30.6)	10 (55.6)	23 (74.2)	0.002
BMI (kg/cm^2^)	24.6 ± 2.9	24.1 ± 3.0	24.2 ± 2.7	0.773
LVEF (%)	61.1 ± 3.9	61.0 ± 2.8	60.4 ± 4.4	0.809
JCSA score	1.1 ± 1.3	3.0 ± 1.0	4.3 ± 1.7	<0.001
Alcohol (%)	6 (16.7)	7 (38.9)	12 (38.7)	0.087
Coronary risk factors (%)				
Smoking	6 (16.7)	3 (16.7)	11 (35.5)	0.144
Hypertension	12 (33.3)	4 (22.2)	7 (22.6)	0.536
Dyslipidemia	16 (44.4)	4 (22.2)	9 (29.0)	0.202
Diabetes mellitus	4 (11.1)	3 (16.7)	4 (12.9)	0.848
Laboratory assessment				
HbA1C	5.6 ± 0.5	5.9 ± 1.0	5.8 ± 0.8	0.614
Creatinine	0.8 ± 0.2	0.8 ± 0.2	0.9 ± 0.2	0.145
Total cholesterol	191.7 ± 49.2	165.9 ± 40.7	172.1 ± 28.3	0.053
LDL-C	116.8 ± 40.2	96.0 ± 29.0	102.4 ± 24.8	0.070
HDL-C	53.4 ± 12.3	47.6 ± 14.0	53.4 ± 12.8	0.248
Triglyceride	141.8 ± 106.3	167.4 ± 106.0	145.9 ± 79.3	0.653
Troponin I peak	0.02 ± 0.02	0.05 ± 0.08	0.14 ± 0.50	0.347

Data are presented as mean ± SD, median (IQR), or number (%). BMI, body mass index; CV, cerebrovascular incident; HDL-C, high-density lipoprotein cholesterol; JCSA, Japanese coronary spasm association; LAD, left anterior descending; LDL-C, low-density lipoprotein cholesterol; LVEF, left ventricular ejection fraction; LCX, left circumflex; MACE, major adverse cardiac event; MI, myocardial infarction; PCI, percutaneous coronary intervention; RCA, right coronary artery.

**Table 2:** Summary of CAG and EG test results.

Variables	Negative (*n* = 36)	Probable Positive (*n* = 18)	Positive (*n* = 31)	*p*
Organic stenosis (%)				0.001
No stenosis	32 (88.9)	16 (88.9)	16 (51.6)	
Insignificant stenosis (25–50%)	4 (11.1)	2 (11.1)	15 (48.4)	
Spasm artery (%)				<0.001
LAD	0 (0)	9 (64.3)	6 (23.1)	
LCX	0 (0)	1 (7.1)	3 (11.5)	
RCA	0 (0)	4 (28.6)	17 (65.4)	
Multi-vessel spasm	0 (0)	1 (5.6)	7 (22.6)	0.006
2 vessels		1 (5.6)	5 (16.1)	
3 vessels		0 (0)	2 (6.5)	

Data are presented as *n* (%). CAG, coronary angiography; EG, ergonovine; LAD, left anterior descending; LCX, left circumflex; RCA, right coronary artery.

**Table 3:** Results and diagnostic performance of CCTA for the detection of vasospastic angina.

	Per Patient	Per Vessel
Results of tests	For patients	For matching/total vessels
Ergonovine		
Total No.	85	197/197
No. of positive + probable positive	49	66/66
No. of negatives	36	131/131
CCTA		
Total No.	85	197/255
No. of positives	31	29/58
No. of negatives	54	168/197
Diagnostic performance of CT	For patients	For matching vessels
Total No.	85	197
No. of true negatives	32	126
No. of true positives	27	24
No. of false negatives	22	42
No. of false positives	4	5
Sensitivity, %	55 (40–69)	36 (25–49)
Specificity, %	89 (74–97)	96 (91–99)
PPV, %	87 (72–95)	83 (66–92)
NPV, %	59 (51–67)	75 (71–78)
Accuracy, %	69 (58–79)	76 (70–82)

Data are presented as number (%). CCTA, coronary computed tomography angiography; NPV, negative predictive value; PPV, positive predictive value.

**Table 4:** Comparison of dual-acquisition MDCTA parameters.

	Negative Group			Positive Group			*p* for CDI
Variables	Baseline CT	IV Nitrate CT	CDI (%)	Baseline CT	IV Nitrate CT	CDI (%)	
Diameter (mm)							
Proximal	3.24 ± 0.65	3.74 ± 0.74	15.8 ± 14.9	2.23 ± 0.58	2.86 ± 0.63	21.4 ± 13.1	0.069
Mid	3.07 ± 0.79	3.53 ± 0.71	17.7 ± 15.4	1.48 ± 0.56	2.60 ± 0.70	42.3 ± 17.3	<0.001
Distal	2.97 ± 0.75	3.47 ± 0.72	15.5 ± 17.0	1.88 ± 0.63	2.58 ± 0.71	26.1 ± 17.9	0.007
Area (mm^2^)							
Proximal	8.61 ± 3.28	11.38 ± 4.31	27.0 ± 25.9	4.24 ± 2.07	6.77 ± 2.85	36.4 ± 20.5	0.066
Mid	7.80 ± 4.04	10.16 ± 3.81	31.5 ± 24.9	1.96 ± 1.53	5.66 ± 2.94	63.6 ± 21.0	<0.001
Distal	7.50 ± 3.73	9.94 ± 3.83	24.9 ± 28.7	3.06 ± 1.84	5.59 ± 2.94	43.2 ± 23.5	0.002

Data are presented as mean ± SD. CCTA, coronary computed tomography angiography; CDI, coronary vessel distensibility index; IV, intravascular.

## Data Availability

Data are the property of the authors and can become available by contacting the corresponding author.

## Supplementary Materials

The following supporting information can be downloaded at: https://www.mdpi.com/article/10.3390/jcm12113753/s1, Table S1: Per patient analysis according to JCSA risk score or clinical/laboratory findings. Table S2: Number of patients and vessels measured for calculating CDI. Table S3. Multi-vessel spasm detection rate of EG test and CCTA in the positive group. Figure S1. Representative case of the first criterion for a positive result on both CCTA and catheterized ergonovine provocation testing. Figure S2: Representative case of the second criterion for a positive result on both CCTA and catheterized ergonovine provocation testing. Figure S3: Correlation between basal coronary tone and coronary spasm constriction detected by CCTA.
