# Supplementary material for: Role of Dual-Acquisition Noninvasive Cardiac CT Imaging for the Detection of Vasospastic Angina
Source: J Clin Med. 2023 May 29;12(11):3753. doi: 10.3390/jcm12113753 (PMC10253957; doi:10.3390/jcm12113753)
Supplement: Supplementary file 1 [file jcm-12-03753-s001.zip › jcm-2337388-supplementary.pdf]

Supplementary Materials

Supplementary Tables

Supplemental Table S1. Per patient analysis according to JCSA risk score or clinical / laboratory findings.

**Category**	**Total data**	**JCSA score**		**Syncope or CPR**	**cTnI ↑**	**ER**	**Alcohol**
		< 3	≥ 3				
Total No.	66	38	28	2	16	15	22
True Negative	24	22	2	0	3	5	6
True Positive	21	6	14	2	10	6	8
False Negative	20	9	12	0	3	4	8
False Positive	1	1	0	0	0	0	0
Sensitivity, %	51	40	54	100	77	60	50
Specificity, %	96	96	100	NC	100	100	100
PPV, %	95	86	100	100	100	100	100
NPV, %	55	71	14	NC	50	56	43

Data are presented as number (%). CPR, cardiopulmonary resuscitation; ER, emergency room; JCSA, Japanese coronary spasm association; NC, non-calculate; NPV, negative predictive value; PPV, positive predictive value.

Supplemental Table S2. Number of patients and vessels measured for calculating CDI.

	Negative group (n=36)	Positive group (n=27)
No. of patients	36	27
No. of vessels	36	24
RCA	21 (58.3%)	14 (58.3%)
LAD	10 (27.8%)	6 (25.0%)
LCX	5 (13.9%)	4 (16.7%)
Spastic type		
Focal		4 (16.7%)
Diffuse		20 (83.3%)

Data are presented as number (%). CDI, coronary vessel distensibility index; LAD, left anterior descending; LCX, left circumflex; RCA, right coronary artery.

Supplemental Table S3. Multi-vessel spasm detection rate of EG test and CCTA in the positive group.

	EG test (n=31)	CCTA (n=31)	p-value
Multi-vessel spasm	7 (22.6)	8 (25.8)	0.767
2 vessels	5 (16.1)	6 (19.4)	
3 vessels	2 (6.5)	2 (6.5)	

Data are presented as number (%). CAG, coronary angiography; EG, ergonovine.

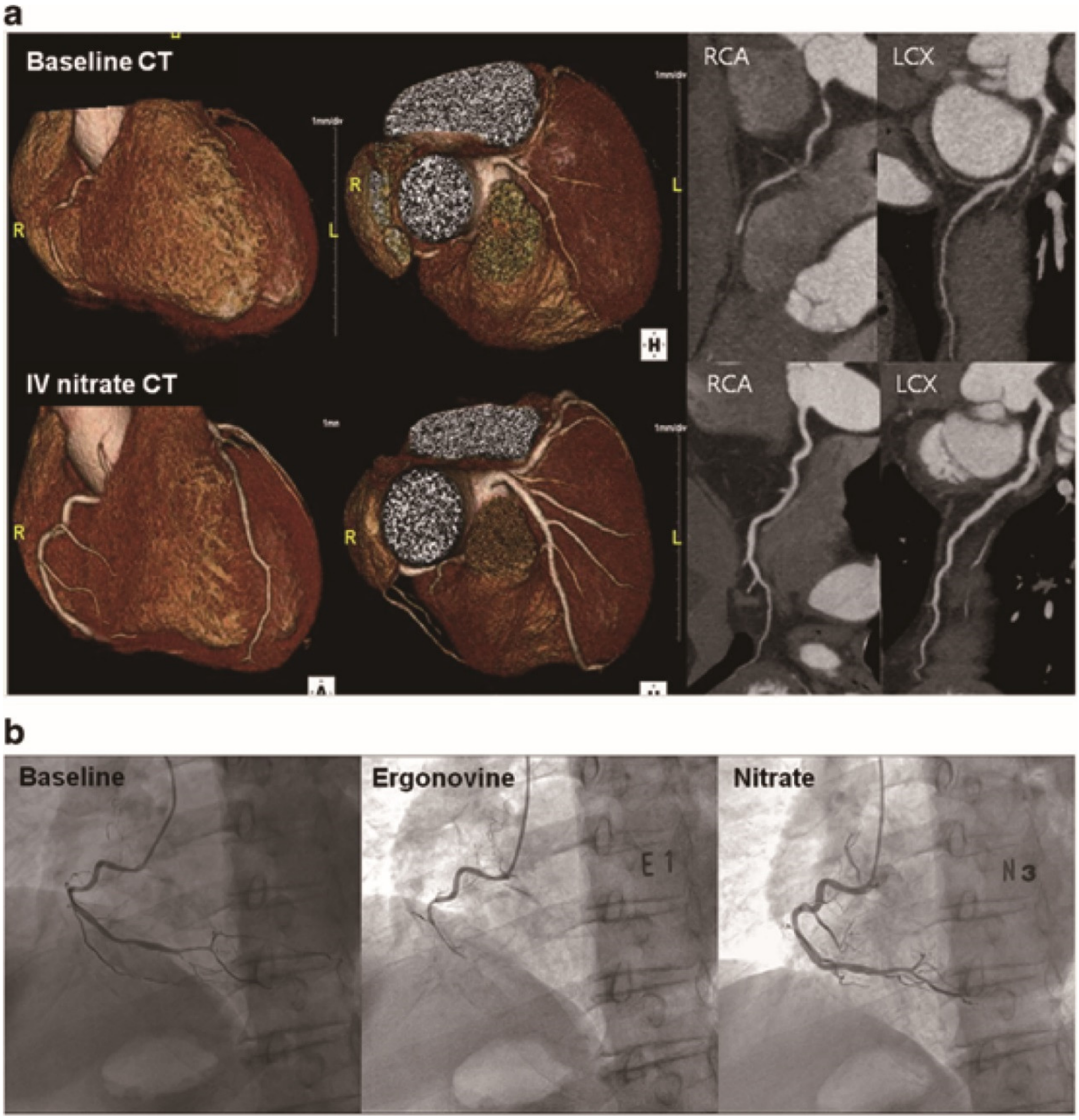

Supplementary Figure S1. Representative case of the first criterion for a positive result on both CCTA and catheterized ergonovine provocation testing.

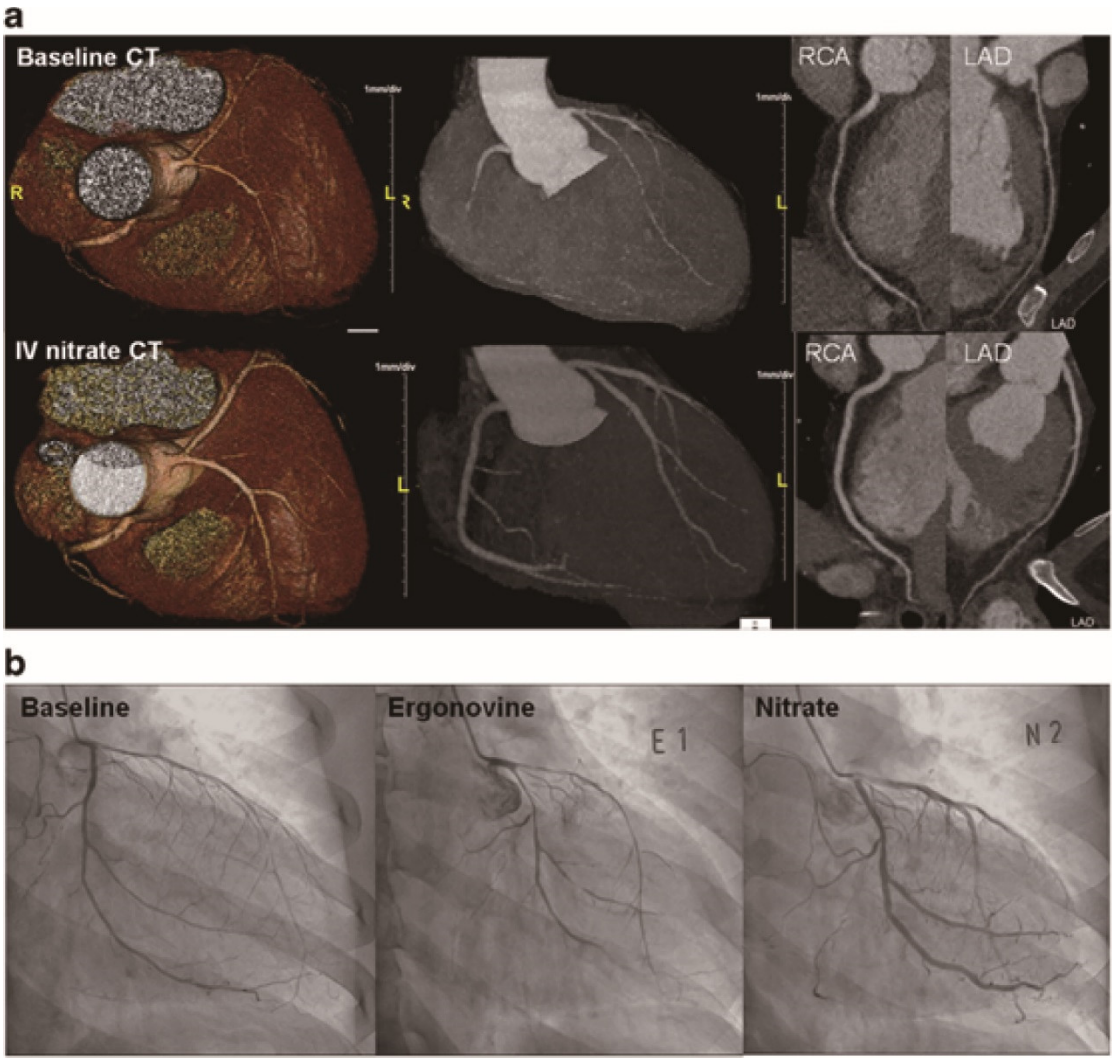

Supplementary Figure S2. Representative case of the second criterion for a positive result on both CCTA and catheterized ergonovine provocation testing.

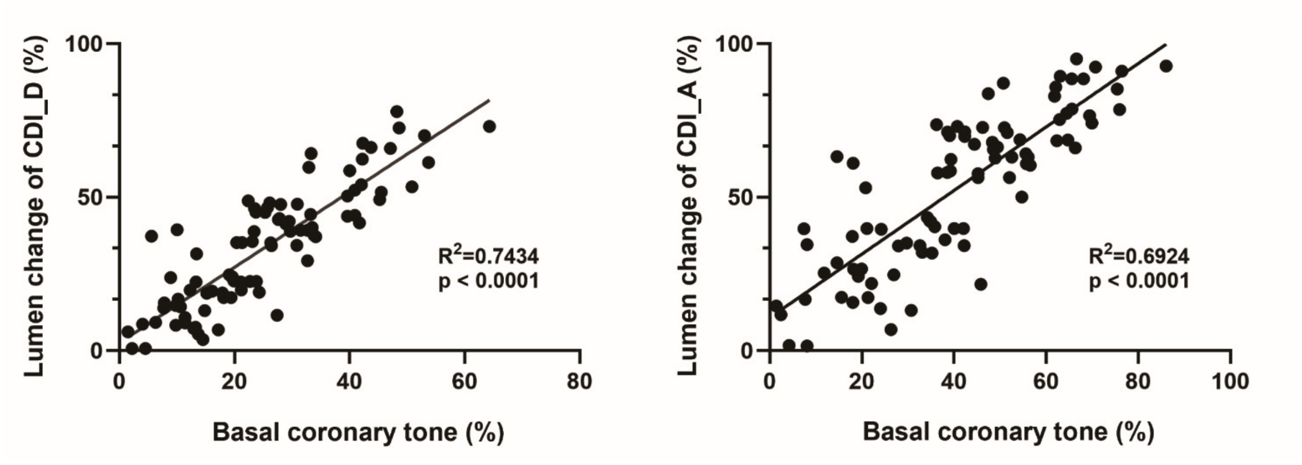

Supplementary Figure S3. Correlation between basal coronary tone and coronary spasm constriction detected by CCTA.
